# A retrospective study on epidemiological analysis of pre-hospital emergency care in Hangzhou, China

**DOI:** 10.1371/journal.pone.0282870

**Published:** 2023-04-18

**Authors:** Jiangang Wang, Yanbin He, Xiaoling Chen, Miaomiao Chen, Chunfu Tang, Fenghua Lu, Ming Qi, Jungen Zhang

**Affiliations:** 1 Hangzhou Emergency Medical Center of Zhejiang Province, Zhejiang, China; 2 DIAN Diagnostics, Hangzhou, China; 3 Key Laboratory of Digital Technology in Medical Diagnostics of Zhejiang Province, Dian Diagnostics Group Co., Ltd., Zhejiang, China; 4 Department of Laboratory Medicine and Department of Obstetrics and Gynecology, Key Laboratory of Reproductive Dysfunction Management of Zhejiang Province, Sir Run Run Shaw Hospital, Zhejiang University School of Medicine, Hangzhou, China; 5 Department of Pathology and Laboratory Medicine, University of Rochester Medical Center, Rochester, New York, United States of America; Azienda Ospedaliero Universitaria Careggi, ITALY

## Abstract

Out-of-hospital cardiac arrest (OHCA) is a leading cause of global mortality, with numerous factors influencing the patient survival rate and prognosis. This study aimed to evaluate the OHCA epidemiology in China and elaborate on the current Hangzhou emergency system status. This retrospective analysis was based on the medical history system of the Hangzhou Emergency Center registered from 2015–2021. We provided a detailed description of OHCA characteristics and investigated the factors affecting the success rate of emergency treatment in terms of epidemiology, causes of onset, bystander rescue, and outcome factors. We included 9585 out-of-hospital cardiac arrest cases, of which 5442 (56.8%) had evidence of resuscitation. Patients with underlying diseases constituted the vast majority (80.1%); trauma and physicochemical factors accounted for 16.5% and 3.4%, respectively. Only 30.4% of patients (about 80.0% of bystanders witnessed) received bystander first aid. The outcome rate of emergency doctors dispatched by emergency centres was significantly higher than doctors dispatched by hospitals. Additionally, physician’s first-aid experience, emergency response time, emergency telephone availability, initial heart rhythm, out-of-hospital defibrillation, out-of-hospital intubation, and using of epinephrine significantly can significantly improve the out-of-hospital return of spontaneous circulation in patients. All steps in pre-hospital care are important for patients, especially for bystander first aid and physician’s first-aid experience. The popularity of first-aid training and the public emergency medical system are not potent enough. We should take those key factors into consideration when developing a pre-hospital care system for OHCA.

## Introduction

Out-of-hospital cardiac arrest (OHCA) refers to the loss of functional cardiac mechanical activity associated with a lack of systemic circulation outside the hospital setting. Typical OHCA symptoms include a sudden stop of the heart and an unexpected abnormal pumping of blood, resulting in insufficient blood supply to the brain, followed by tissue damage in the brain, heart, and kidney [1]. OHCA is one of the most serious acute diseases with high mortality risk [2]. The average global OHCA incidence among adults is 55 per 100,000 people annually [3]; the overall survival rate is between 0.6% and 25% [4]. In Europe and the United States, approximately 275,000 and 155,000 adult patients, respectively, with OHCA receive emergency medical treatment each year, with a survival rate of 8–10% [5]. In China, approximately 550,000 people experience cardiac arrest (CA) yearly; however, the OHCA survival rate is less than 1% [3], which is far from that in countries with an emergency medical rescue service system. Therefore, it is important to improve OHCA treatment capacity and optimise its response system in China.

The 2015 American Heart Association (AHA) guidelines for Cardiopulmonary resuscitation (CPR) [6] suggest that pre-hospital first aid for patients with OHCA is the key initiator in the "chain of survival,” including cardiac arrest identification, emergency assistance, CPR initiation. There is only 10 min, termed “golden 10 minutes,” to rescue a patient with OHCA in the pre-hospital first aid stage. The patient’s chance of survival drops by 7–10% for every minute of delay in receiving CPR and defibrillation after collapsing [7]. Therefore, pre-hospital care is critical for the survival of patients with OHCA. In the “chain of survival,” immediate recognition of cardiac arrest and activation of the emergency response system activation and CPR initiation are mainly performed by bystanders [1,8,9]. Improving the medical treatment capacity of bystanders to treat patients with OHCA will build a line of defence to ensure patients with OHCA’s survival and prognosis. Thus, bystander first-aid is a medical intervention behaviour in which the eyewitness initiates CPR before professional medical staff arrival, shortening the treatment time and improving treatment effects [10]. Besides, some studies reported that emergency response time, emergency telephone availability, patients’ initial heart rhythm type, and out-of-hospital defibrillation are key factors that increase the survival rate [1,9,11–13]. In addition, the composition of pre-hospital emergency physicians may be another critical issue. Professional pre-hospital first aid teams often show quick emergency response and good cooperation and communication skills compared to unprofessional teams.

Hangzhou is a typical provincial capital city in China, with a total population of about 11.93 million permanent residents. Hangzhou Emergency Center has set up 4 first-aid stations and 30 first-aid points in the urban area. The training of international resuscitation guidelines [14] has been popularized in the field of emergency care, however, the success rate of pre-hospital resuscitation in China has been very low and Hangzhou is far more than average but still low compared with the world. which can be associated with the poor prognosis and unpredictable time and place of cardiac arrest. More importantly, it is closely related to all steps of pre‐hospital emergency service and analysing clinical pathways to find deficiencies can help improve the success rate of pre-hospital resuscitation.

In this study, we collected cases of OHCA in the Hangzhou Emergency Center from 2015–2021 and analysed the current situation in the OHCA pre-hospital emergency medical treatment system regarding epidemiology, onset time, causes of onset, bystander rescue, and outcome factors. This study aimed to provide a reference for improving OHCA pre-hospital care capability in China.

## Methods

### Study design and settings

All pre-hospital emergency medical record data between January 2015 and December 2021 were retrieved from the database of Hangzhou Emergency Center, Zhejiang Province. We obtained the patients’ age, sex, and primary disease history from the OHCA registry records. Data related to cardiac arrest were collected, including time of symptom onset, pre-hospital emergency response time, first bystander rescue, death cause, medical treatment by on-site medical staff (drug use, endotracheal intubation, and electric defibrillation), patient outcomes (spontaneous circulation, spontaneous breathing, and consciousness recoveries), and rescue information provided by family members. Missing data were recorded truthfully as “unknown” for the gathered patient history. Besides, inclusion criteria for resuscitation were patients with a sudden loss of consciousness and absence of carotid pulse, patients with signs of cyanosis apnoea and ECG findings indicating ventricular arrest without a pulse, patients with electrical activity and ventricular fibrillation, and patients with cardiac and respiratory arrest within 30 min. Exclusion criteria for resuscitation were patients with cardiac arrest duration of >30 min, patients with rigor mortis and lividity, and patients with disfigured head and trunk.

### Statistical analysis

A patient information database was established using Excel, and the data were processed using R software (version 4.1.2). The normal distribution measurement data were expressed as the mean ± SD, and the count data were expressed as the number of cases (rate). Spontaneous circulation recovery was used as the standard to evaluate the patients with OHCA’s prognosis. The χ^2^ test was performed for all factors that might affect the prognosis; a P-value <0.05 was considered statistically significant.

### Ethics

Retrospective study is not mainly concerned with the disassociation of information about an individual, thus the need for consent was waived by the ethics committee. The ethical approval of this study came from the Ethics Committee of Hangzhou Emergency Medical Center (No: HZEMC-2020-09).

## Results

### Patient characteristics

**Fig 1:**
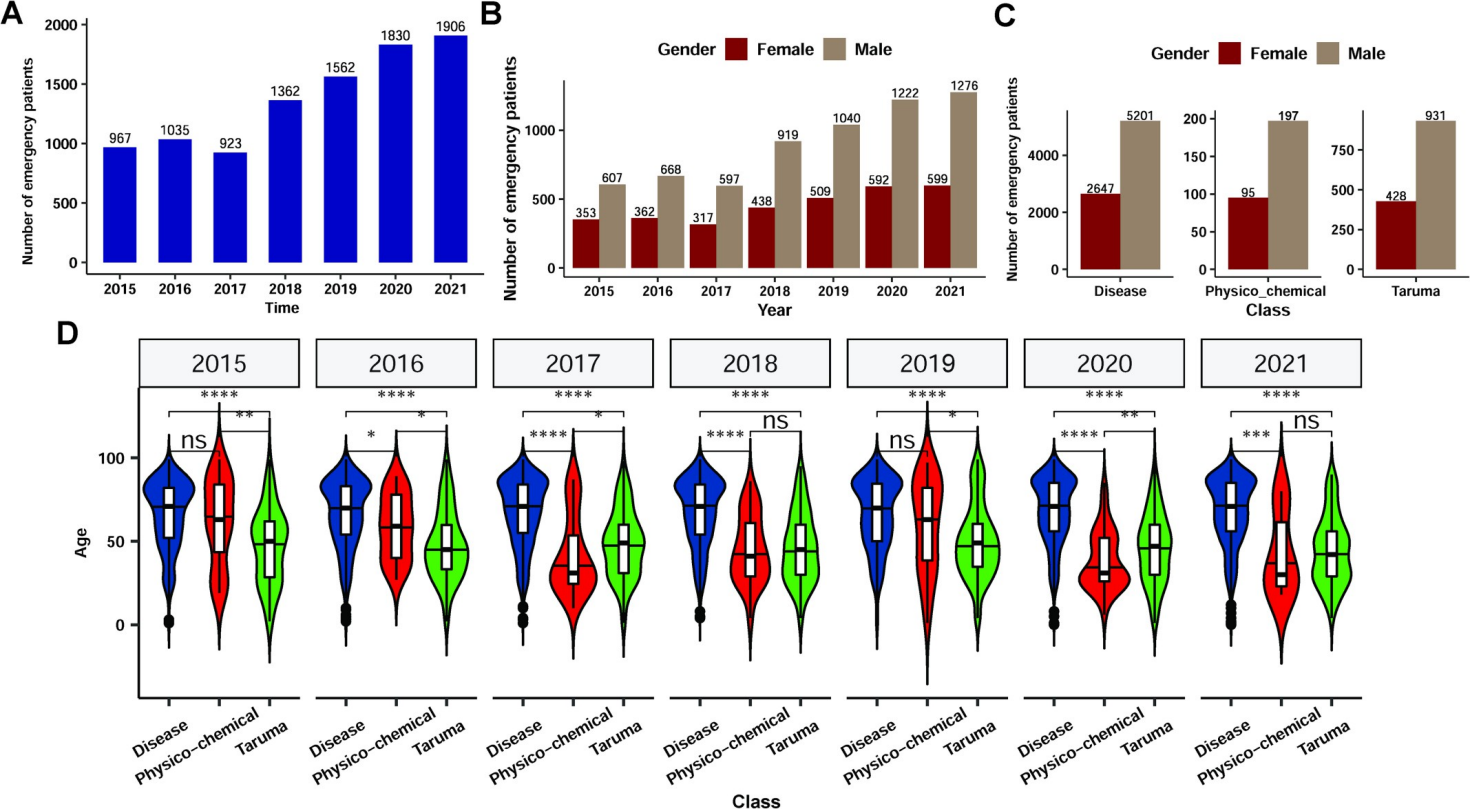
Demographic information of emergency patients with cardiac arrest. The number of emergency patients with cardiac arrest is indicated yearly (2015–2021) (A). The number of emergency patients with cardiac arrest by sex is indicated yearly (2015–2021) (B). The number of emergency patients by sex among the different causes of cardiac arrest, including disease, physicochemical stimulation, and trauma (C). Age distribution of patients with cardiac arrest due to different causes (disease: Blue, physicochemical: Red, trauma: Green) is indicated yearly (D) (* p < 0.05, ** p < 0.01, *** p < 0.001, **** p < 0.0001).

We collected data from 9,585 patients with cardiac arrest who were treated at the emergency center in Hangzhou between 2015 and 2021 (Table 1). The patients’ median age was 64 years (IQR, 50–83 years). Data analysis over the years showed an annual increase in the number of patients with cardiac arrest (Fig 1A), and the number of male patients was approximately twice as high as that of female patients (Fig 1B). Analysis results of the causes of cardiac arrest revealed that 80.1% (n = 7911) patients experienced cardiac arrest due to their primary diseases, 16.5% (n = 1381) due to trauma, and 3.4% (n = 293) due to physicochemical stimulation. The median age of patients with cardiac arrest induced by underlying diseases, trauma, and physicochemical stimulation was 67 years (IQR, 55–84 years), 46 years (IQR, 30–60 years), and 48 years (IQR, 29–67 years), respectively, with episodes occurring mainly at home and in public places. The analysis of the three different causes of cardiac arrest (underlying diseases, physicochemical factors, and trauma) showed that the proportion of male patients was higher than that of females (Fig 1C). In addition, the ages of patients with cardiac arrest due to primary diseases were significantly higher than those due to trauma and physicochemical factors in each year (* p <0.05, ** p <0.01, *** p < 0.001, **** p <0.0001) (Fig 1D).

**Table 1:** Epidemiological variables of the study population.

Epidemiological features	Total	Disease	Trauma	Physicochemical factors
**Patients n (%)**	9585 (100%)	7911 (80.1%)	1381 (16.5%)	293 (3.4%)
**Sex n (%)**				
** Male**	6329 (66.03%)	5201 (65.74%)	931 (67.41%)	197 (67.24%)
** Female**	3170 (33.07%)	2647 (33.46%)	428 (30.99%)	95 (32.42%)
**Age (Mean + IQR)**	64 (50–83)	67 (55–84)	46 (30–60)	48 (29–67)
**Location n (%)**				
** Home**	5672 (59.18%)	5325 (67.31%)	201 (14.55%)	146(49.83%)
** Public place**	1236 (12.90%)	731 (9.24%)	434 (31.43%)	71 (24.23%)
** Workplace**	445 (4.64%)	312 (3.94%)	117 (8.47%)	16 (5.46%)
** Traffic**	612 (6.38%)	229 (2.89%)	382 (27.66%)	1 (0.34%)
** Medical institution**	222 (2.32%)	201 (2.54%)	16(1.16%)	5(1.71%)
** Other people**	1398 (14.59%)	1113 (14.07%)	231 (16.73%)	54 (18.43%)
**The initial ECG n (%)**				
** VF/VT**	528 (5.51%)	484 (6.12%)	39 (2.82%)	5 (1.71%)
** PEA**	359 (3.75%)	293 (3.70%)	61 (4.42%)	5 (1.71%)
** Ventricular standstill**	6229 (65.71%)	5200 (65.73%)	891 (64.52%)	138 (70.65%)
** Other**	2400 (25.04%)	1934 (24.45%)	390 (28.24%)	76 (25.93%)
**Resuscitable patient n (%)**	5442 (56.78%)	4630 (58.53%)	672 (48.66%)	140 (47.78%)
**Feeble respiration**	347 (3.62%)	307 (3.88%)	33 (2.39%)	7 (2.39%)
**Response time (min, mean ± sd)**	12.9±8.8	12.71±8.7	13.87±9.7	13.38±7

Note: Data are shown as mean ± SD.; VF, ventricular fibrillation; VT, ventricular tachycardia; PEA, pulseless electrical activity.

Cardiac arrest is the final manifestation of most acute and critical clinical conditions. Whether the initial rhythm of a patient with cardiac arrest is shockable is critical to the patient’s resuscitation success and prognosis. Shockable rhythms include ventricular fibrillation (VF) and pulseless ventricular tachycardia (VT), while non-shockable rhythms include pulseless electrical activity (PEA) and asystole (ASY). In this study, there were 528 (5.51%) shockable rhythms and 6,588 (68.73%) non-shockable rhythms, including 359 (3.75%) PEA and 6,229 (65.71%) ASY cases. A total of 5,442 patients (56.8%) had resuscitation indications, including unconsciousness, absence of respiration, or only agonal breathing, with the disappearance of large artery pulsation; whereas, 4,143 patients (43.2%) who presented with cadaveric lividity, head and trunk disfigurement, and cardiac arrest lasting for more than 30 min were excluded. The emergency response time was (12.9±8.8) min.

### The time of onset of cardiac arrest

**Fig 2:**
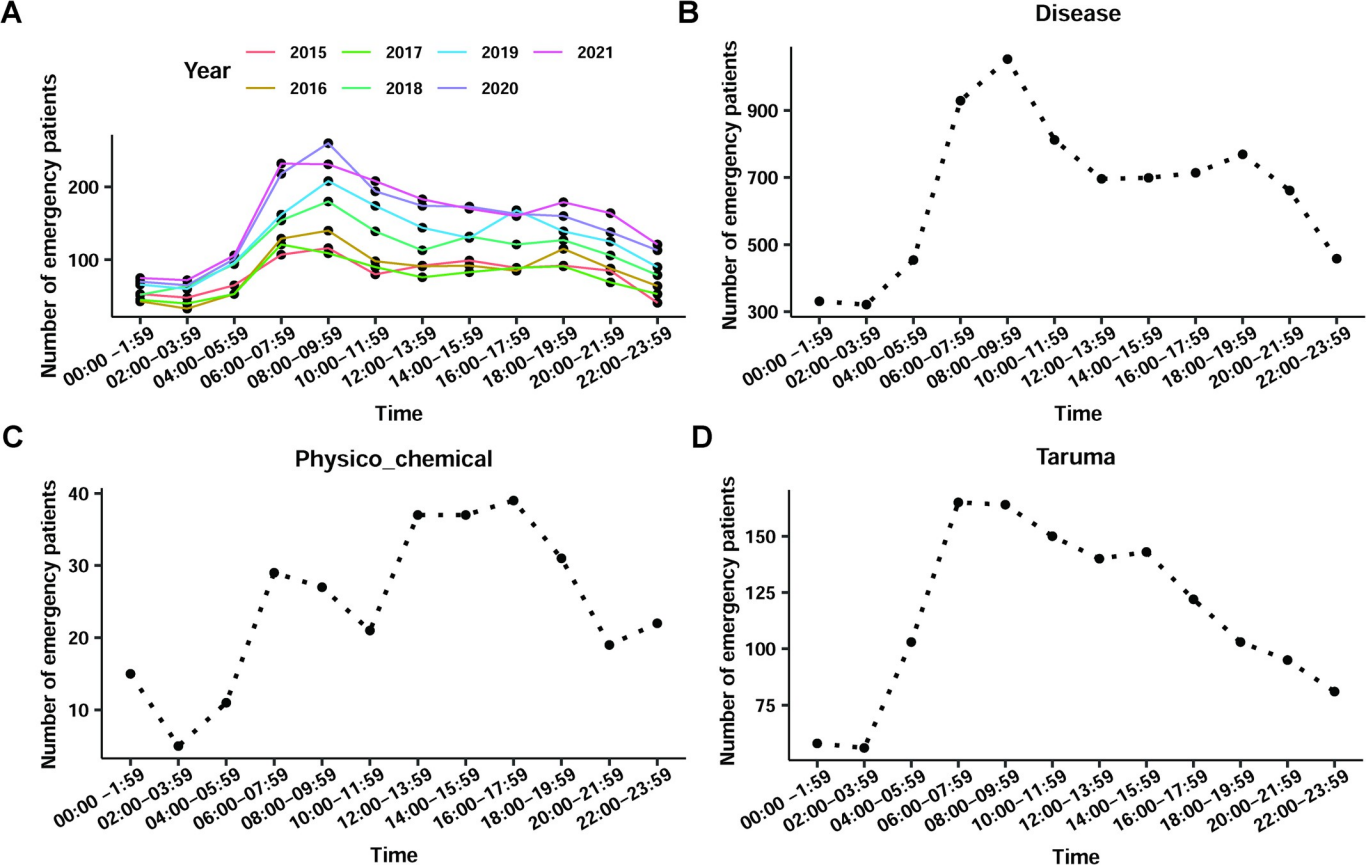
Analysis of onset time in patients with cardiac arrest. The number of occurrences in emergency patients with cardiac arrest at different times daily (different colored lines represent different years) (A). And the number of occurrences in emergency patients with cardiac arrest at different times attributable to disease (B), physicochemical causes (C), and trauma (D) daily.

By analysing the frequency of calls for help that were received by the emergency centre in each period for one day, it was found that the frequency was highest between 6:00–7:59 and 8:00–9:59, which was higher than that between 4:00–5:59, and decreased after 10:00, dropping to a minimum after midnight (0:00). This trend remained largely consistent from 2015 to 2021 (Fig 2A). On dividing the causes of cardiac arrest, it was found that the number of calls was highest between 6:00 and 10:00 for patients with underlying diseases, with a gradual downward trend after 10:00. The lowest number of calls was in the early hours of the day, consistent with the overall results (Fig 2B). Cardiac arrest caused by physicochemical stimulation occurred more often during the day, with the highest number of calls occurring in the afternoon (between 12:00 and 18:00) (Fig 2C). Traumatic cardiac arrests tended to occur during the morning rush hour (between 6:00 and 10:00), after which the number of calls for help continued to decline (Fig 2D). In addition, cardiac arrest incidence was seasonally related, with patients with underlying diseases having a higher incidence in spring and winter and those with cardiac arrest due to physicochemical stimulation or trauma having a higher incidence in summer (S1 Fig).

### Analysis of the causes of cardiac arrest

**Fig 3:**
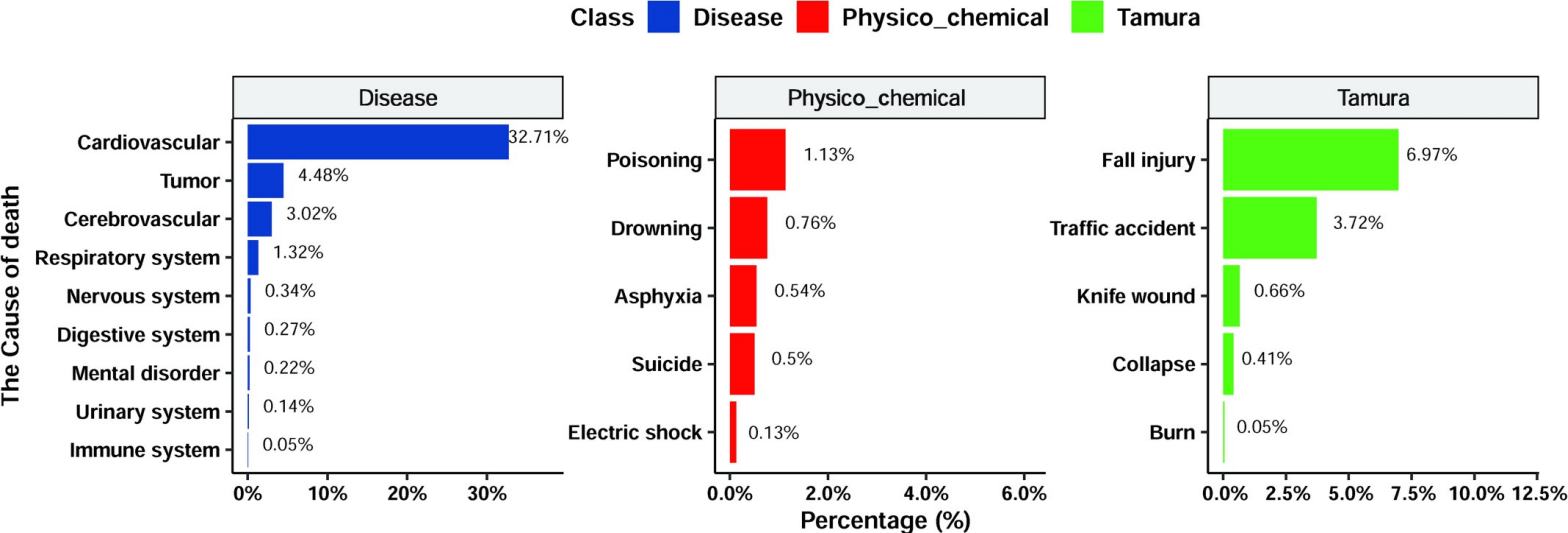
Analysis of causes of death in different types of patients with OHCA. Death proportions in cardiac arrest due to disease (left), physicochemical causes (middle), and trauma (right).

There were several causes of cardiac arrest (S1 Table). It was found that 80.1% of cardiac arrests were due to underlying diseases, with the diseases related to cardiovascular, cerebrovascular, and respiratory systems, and presence of tumours accounting for the largest proportion, at 32.71%, 3.02%, 1.32%, and 4.48%, respectively. Traumatic cardiac arrest accounted for 16.5% of patients overall, with fall injuries, traffic accidents, knife injuries, and collapse crush injuries accounting for the most deaths at 6.97%, 3.72%, 0.66%, and 0.41%, respectively. The proportion of cardiac arrests due to physicochemical stimulation was only 3.4%; poisoning, drowning, and asphyxiation accounted for the highest number of cardiac arrests at 108 (1.13%), 73 (0.76%), and 52 (0.54%), respectively (Fig 3).

#### Bystander rescue

**Fig 4:**
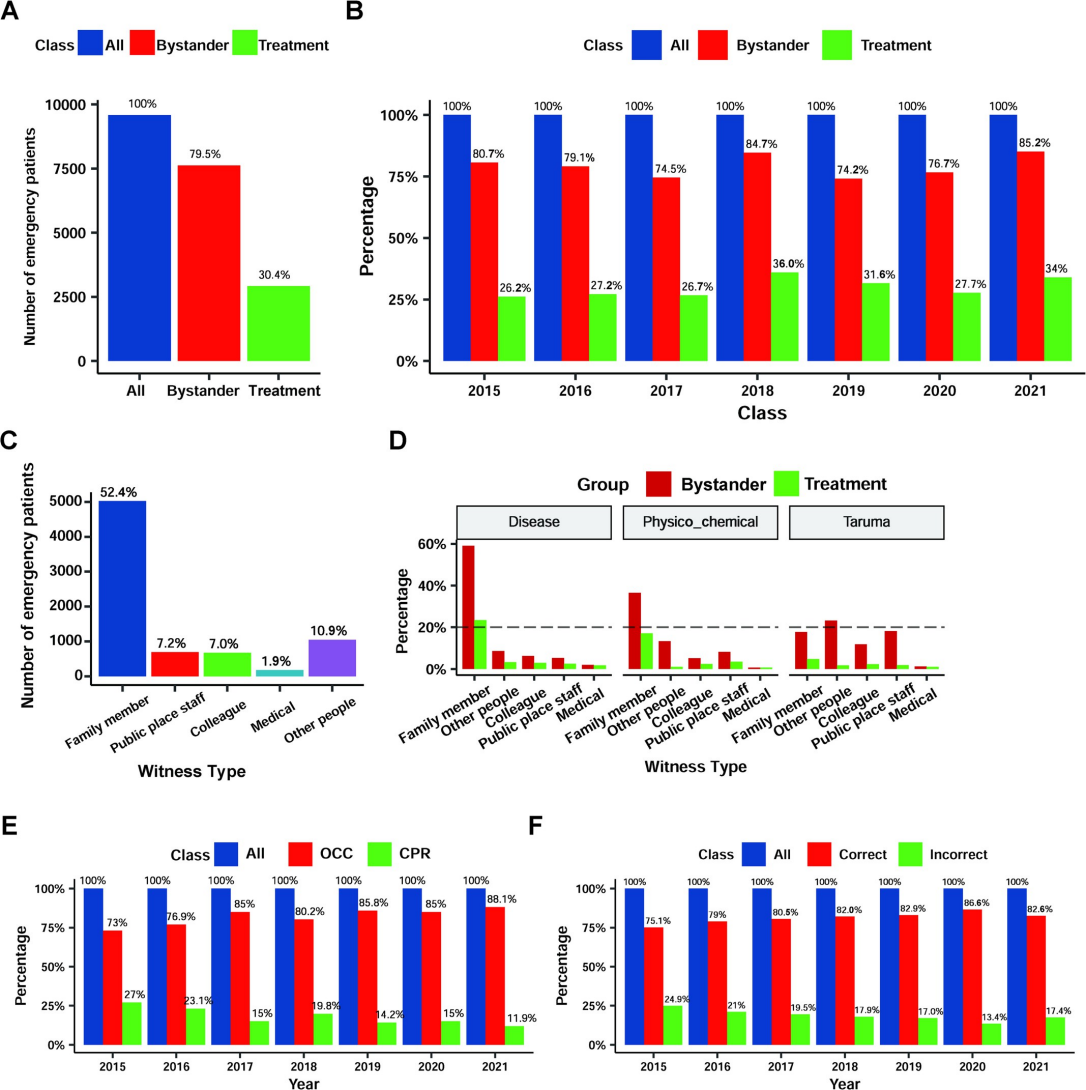
Analysis of on-site treatment of bystanders. The proportion of bystanders and bystanders who performed first aid during cardiac arrest is indicated for overall (A) and each year (2015–2021) (B). The blue bars represent the percentage of all patients, the red bars represent the percentage of bystanders, and the green bars represent the percentage of treatment. Proportion of different types of people in bystanders (C). The proportion of different types of witnesses in different causes of cardiac arrest and the proportion of treatment taken (D). Proportion of all treatments using OCC (red) and CPR (green) each year (2015–2021) (E). OCC: Open chest cardiac compression. CPR: Cardiopulmonary resuscitation. Correct rate (red) and incorrect rate (green) of rescue by bystanders (F).

Bystander rescue is an important part of pre-hospital emergency care. Most patients had bystanders during cardiac arrest, with 7,608 cases, accounting for 79.5% of all cases. The proportion of bystanders performing lifesaving treatment was relatively low (30.4%) (Fig 4A). The percentage of bystander-witnessed cardiac arrests from 2015 to 2021 ranged from 74.2% to 85.2%; the highest was in 2021 (accounting for 85.2%) (Fig 4A). However, an analysis of the data on bystanders performing lifesaving treatment each year showed that the proportion of bystanders performing lifesaving treatment was relatively low (between 25% and 36%). Similarly, a longitudinal comparison showed that the population of on-scene bystanders performing lifesaving treatment increased from 2015 to 2021, with the percentage increasing from 26.2% to 36% (Fig 4B). An analysis of the different types of patients with cardiac arrest showed that the proportion of bystanders performing rescue was relatively low, which had been increasing over the years (S2 Fig).

Among bystanders, 52.4% were family members, 7.2% were public workers, 7% were colleagues, and 1.9% were professionals (Fig 4C). The bystanders of emergency patients who experienced cardiac arrest due to underlying diseases were mainly family members, accounting for 59%, and the proportion of rescue efforts by family members was 23.4%. The other bystanders had a lower rate of first-aid use. For patients who suffered cardiac arrest by physicochemical stimulation, bystanders were also mainly family members, accounting for 36.5%, with their rescues accounting for 17%. The bystander population in traumatic cardiac arrests was relatively uniform, with staff who work in public places accounting for 18.1%, family members accounting for 17.6%, and colleagues accounting for 11.8%. The percentage of these populations performing on-site first aid was generally low (<5%). Among the bystanders on the scene, professional medical personnel accounted for a relatively low percentage (1–2%), but all medical personnel provided on-site first aid to patients (Fig 4D).

In bystander rescue, only external chest compressions were performed in 2,448 cases (25.5%), complete CPR was performed in 440 (4.6%), and open-chest cardiac compression (OCC) was used by 73–88% of bystanders, which increased by 13% in 2021 compared with 2015. The proportion of bystanders using CPR was relatively low, accounting for 10–30% (Fig 4E). About 70–80% of the rescuers were able to give correct first aid at the scene. Since 2015, the rate of correct bystander rescue has increased annually. By 2021, the accuracy rate had increased from 75% to 82%, and the rescue accuracy rate in 2020 was 86.6%. (Fig 4F).

### Factors influencing the outcome of patients in cardiac arrest

Excluding the patients with cadaveric lividity, head trunk disfigurement, and cardiac arrest for more than 30 min, 5,442 cardiac arrest patients with resuscitation indications were analysed; 414 had a ROSC and 153 (2.81%) had recovered sanity (S2 Table). Combining multiple factors, the results showed that telephone CPR, bystander first aid, patient’s initial ventricular fibrillation or tachycardia rhythm, out-of-hospital defibrillation, type of emergency care practitioner, hospital-physician continuous emergency care duration, out-of-hospital tracheal intubation, out-of-hospital use of epinephrine, and emergency response time significantly improved out-of-hospital ROSC in patients with OHCA, further improving patient survival rate after hospital admission (Table 2).

**Table 2:** Influencing factors of ROSC in out-of-hospital cardiac arrest patients.

Factor		Resuscitation rate [%(n/n)]	χ2	P-value
**Age**	≥65	7.29% (208/2853)	1.21	0.26
	<65	8.11% (192/2365)		
**Telephone CPR**	Yes	8.70% (223/2563)	8.24	**0.004**
	No	6.63% (191/2879)		
**Bystander first aid**	Yes	9.65% (240/2486)	16.21	**< 0.001**
	No	6.54% (163/2492)		
**Emergency care practitioners**	Emergency Specialist	11.35% (186/1638)	46.83	**< 0.001**
	Hospital-dispatched Physicians	5.99% (228/3804)		
**Hospital-dispatched physician’s work time (month)**	< = 6	4.77% (141/2953)	34.8	**< 0.001**
	>6	10.2% (87/851)		
**Initial heart rhythm** **VT/VF**	Yes	26.1% (85/326)	143.5	**< 0.001**
	No	6.58% (304/4326)		
**Out-of-hospital defibrillation**	Yes	12.72% (156/1226)	58.94	**< 0.001**
	No	6.12% (258/4216)		
**Out-of-hospital tracheal intubation**	Yes	17.94% (165/935)	146.23	**< 0.001**
	No	5.11% (144/2820)		
**Out of hospital use of epinephrine**	Yes	11.77% (227/1929)	67.53	**< 0.001**
	No	5.04% (127/2520)		
**Responses time** **≤ 10 min**	Yes	8.66% (206/2378)	6.69	**< 0.001**
	No	6.79% (208/3064)		

The p value of significantly differed clinical indexes are displayed in bold. Note: VF, ventricular fibrillation; VT, ventricular tachycardia.

## Discussion

This is a register-based, retrospective observational study that collected detailed emergency information on 9,585 patients with OHCA in Hangzhou Emergency Center from 2015 to 2021 to analyse the current status of pre-hospital emergency medical services (EMS). This analysis found that the proportion of OHCA in men was approximately two-fold higher than in women. Several risk factors such as higher life stress, lack of exercise, high-calorie diet, long-term smoking, and alcohol consumption in men may be responsible. In addition, diseases account for approximately 80% of OHCA cases, with cardiovascular diseases accounting for 30–40%, mostly in middle-aged and older adults. Middle-aged and older adults are often accompanied by chronic diseases and symptoms may be aggravated when stimulated by adverse incentives such as overwork, heavy drinking, mood swings and constipation, which is a significant cause of death in patients. We also confirmed that OHCA occurred more frequently between 6:00 and 10:00 at home. Morning blood pressure elevation increases a patient’s susceptibility to sudden death. And falling asleep at night is not easy to detect in patients. There are very few patients with OHCA in medical institutions where healthcare facilities are well equipped and favourable for timely diagnosis and treatment.

Current evidence suggests that bystander first aid significantly improves the pre-hospital ROSC for patients with OHCA. Our study revealed that only 30.4% of patients (about 80% of bystander witnessed) received bystander first aid. We speculated that there may be the following reasons. More than half of the bystanders were families and specialist healthcare workers who were non-professionals and lacked first-aid training or awareness. This contributed to patients not being rescued effectively during the key period. Besides, OHCA has a golden rescue time of 10 min after cardiac arrest onset [15]. Bystanders cannot easily recognize cardiac arrest symptoms, and commonly mistake it for syncope and seizure, especially for patients with a persistent wheeze for several minutes [16]. In addition, we found that the bystander CPR rate was lower. Previous reports demonstrated [3,17] that bystander CPR rates were 46.1% in the US, 29% in Canada, 32.2% in Japan, and 21.1% in Austria. There might be several reasons for the lower CPR rate, including fear of harming patients and reluctance to perform mouth-to-mouth breathing [1]. And telephone CPR is a well-established determinant factor related to ROSC. Resuscitation guidelines [14] suggest that the bystanders first make a phone call to report an emergency for OHCA recognition and oral guidance by dispatchers, which can help bystander CPR assistance rescue people suspected of having cardiac arrest with loss of consciousness or abnormal respiration. Considering the low bystander CPR rate in China, we suggest that the relevant authorities should introduce relevant emergency policies to encourage bystander first aid. Also, first-aid training should be extended to the wider population to promote public awareness and practical skills, helping people overcome the psychological and operational obstacles in performing first aid.

We found that the outcome rate of emergency doctors dispatched by emergency centres was significantly higher than that of doctors dispatched by hospitals. Previous investigations have found that the resuscitation success rate of emergency physicians was 31.7%, higher than that of doctors dispatched by hospitals. This indicated that emergency physicians were more experienced, competent, and had advanced emergency skills in first-aid care. They reduced the variability of the treatment process and shortened the emergency time. In addition, hospital-dispatched physicians had been in the emergency department for over six months, which improved the ROSC rates of patients with OHCA. Therefore, with the medical conditions in hand, we suggest that local emergency medical service increase the proportion of emergency physician dispatches and enhanced training hospitalists in first aid. Alternatively, hospital-dispatched physicians’ first-aid work time should be maintained for six months.

Additionally, current evidence suggests that effective measures, including pre-hospital endotracheal intubation or epinephrine treatment, significantly improve pre-hospital ROSC in patients with OHCA. Out-of-hospital endotracheal intubation is usually performed by professional emergency physicians with rich experience in emergency care and skilled CPR techniques, which are more conducive to patient recovery [18,19]. Previous studies have also shown that treatment with epinephrine is associated with an increased survival rate in patients with OHCA [20]. However, some researchers have found that epinephrine may impair cerebral blood flow, increase ventricular arrhythmia, and induce myocardial dysfunction after ROSC [21]. Therefore, further investigation of the correlation between epinephrine dosage and duration of treatment is needed.

## Limitations

Our study had several limitations that need to be acknowledged. This study belongs to the retrospective research on OHCA that only recorded pre-admission information of patients. OHCA can be sudden and unexpected in a complicated external environment; some device and medical treatment was not suitable for field deployable. Also, there is different health care subjects between pre-hospital and in-hospital. Therefore, our study could not access information of rescue situation after patients entering to the hospital, such as emergent coronary angiography, extra-corporeal membrane oxygenation. Besides, information records and management were not standardized due to the study’s retrospective nature. Its limited follow-up information led to some missing information and bias with a long follow-up period. Moreover, the representability might be insufficient as emergency data were collected solely in the Hangzhou area, with the health care level varying considerably between regions in China; therefore, the conclusion cannot represent the situation in other Chinese regions. These limitations should be further confirmed by conducting large-scale prospective studies.

## Conclusions

In conclusion, all steps in pre-hospital care are important for patients, especially for bystander first aid and emergency physician’s experience. While the popularity of first-aid training and the public emergency medical system are not potent enough, and also lack emergency skills and relevant first-aid knowledge in our population. Therefore, it is suggested that professional groups and institutions strengthen the publicity of first aid’s relevant knowledge through legal, cultural, training, and increase the proportion of emergency physician dispatches, resulting in more effective patient treatment.

## Supporting information

S1 Fig The number of occurrences in emergency patients with different causes in different seasons (Q1, Q2, Q3, and Q4). (TIF)

S2 Fig The proportion of bystanders treated in patients with cardiac arrest attributable to disease, physicochemical causes, and trauma each year (2015–2021). (TIF)

S1 Table Causes of death in the study population. (DOCX)

S2 Table Outcome population statistics of the study population. (DOCX)

S1 File (XLSX)
