# Supplementary figures and images for: A retrospective study on epidemiological analysis of pre-hospital emergency care in Hangzhou, China
Source: PLoS One. 2023 Apr 18;18(4):e0282870. doi: 10.1371/journal.pone.0282870 (PMC10112809; doi:10.1371/journal.pone.0282870)

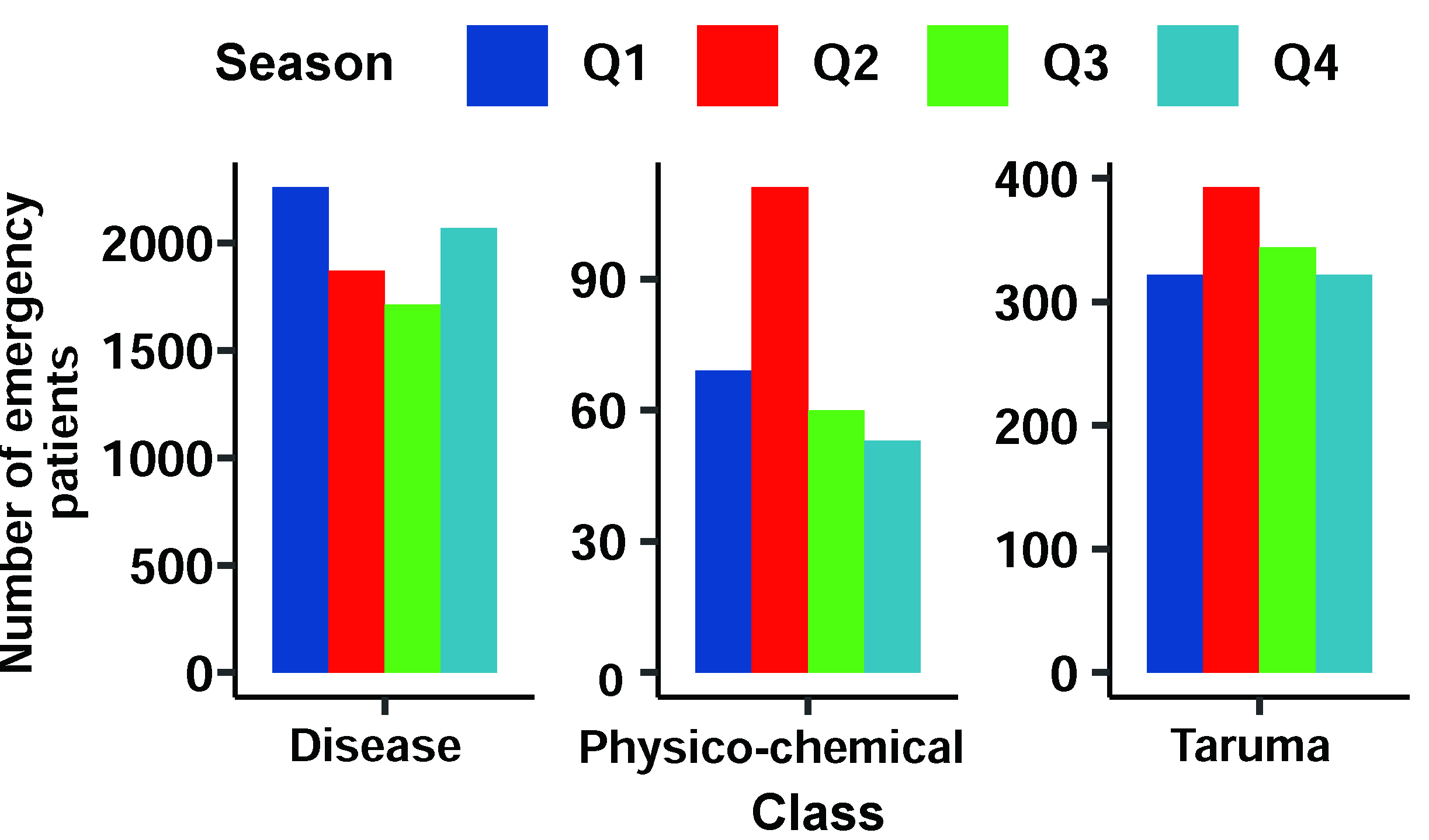

Supplement: S1 Fig — (TIF) [file pone.0282870.s001.tif]

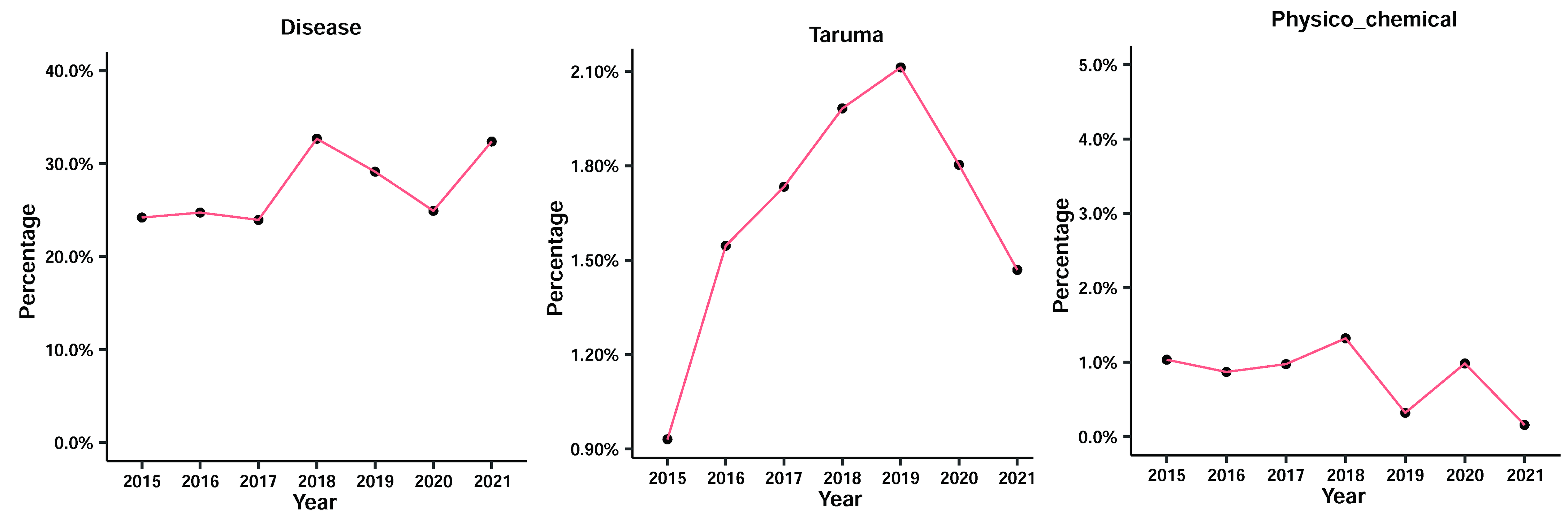

Supplement: S2 Fig — (TIF) [file pone.0282870.s002.tif]
