# Supplementary material for: A retrospective study on epidemiological analysis of pre-hospital emergency care in Hangzhou, China
Source: PLoS One. 2023 Apr 18;18(4):e0282870. doi: 10.1371/journal.pone.0282870 (PMC10112809; doi:10.1371/journal.pone.0282870)
Supplement: S1 Table — (DOCX) [file pone.0282870.s003.docx]

**S1 Table. Causes of death in the study population**

|  | **Death Cause** | **Number** | **Percentage (%)** |
| --- | --- | --- | --- |
| **Disease** | Cardiovascular | 3135 | 32.71% |
|  | Tumor | 429 | 4.48% |
|  | Cerebrovascular | 289 | 3.02% |
|  | Respiratory system | 127 | 1.32% |
|  | Nervous system | 33 | 0.34% |
|  | Digestive system | 26 | 0.27% |
|  | Mental disorder | 21 | 0.22% |
|  | Urinary system | 13 | 0.14% |
|  | Immune system | 5 | 0.05% |
|  | Other Diseases | 3833 | 39.99% |
| **Trauma** | Fall injury | 668 | 6.97% |
|  | Traffic accident | 357 | 3.72% |
|  | Knife wound | 63 | 0.66% |
|  | Collapse | 39 | 0.41% |
|  | Burn | 5 | 0.05% |
|  | Other Trauma | 249 | 2.60% |
| **Physico-chemical factors** | Poisoning | 108 | 1.13% |
|  | Drowning | 73 | 0.76% |
|  | Asphyxia | 52 | 0.54% |
|  | Suicide | 48 | 0.50% |
|  | Electric shock | 12 | 0.13% |
