# Supplementary material for: A retrospective study on epidemiological analysis of pre-hospital emergency care in Hangzhou, China
Source: PLoS One. 2023 Apr 18;18(4):e0282870. doi: 10.1371/journal.pone.0282870 (PMC10112809; doi:10.1371/journal.pone.0282870)
Supplement: S2 Table — (DOCX) [file pone.0282870.s004.docx]

**S2 Table. Outcome population statistics of the study population**

| **Outcome** | **Patients [ n (%)]** |
| --- | --- |
| Recovery of spontaneous circulation (ROSC) | 414 (7.61%) |
| Cerebral resuscitation | 153 (2.81%) |
